# The Effects of Sleeper and Superman Stretches on Time-Zero Shoulder Range of Motion in Collegiate Athletes

**DOI:** 10.7759/cureus.22600

**Published:** 2022-02-25

**Authors:** Amr Tawfik, Gregory R Toci, Francis Sirch, Brian Gibbs, Evan Conte, Daniel Fletcher, Joshua Hornstein, Christopher Aland

**Affiliations:** 1 Hand Surgery, Rothman Orthopaedic Institute, Philadelphia, USA; 2 Orthopaedic Surgery, Rothman Orthopaedic Institute, Philadelphia, USA; 3 Orthopaedics, Rothman Orthopaedic Institute, Philadelphia, USA; 4 Sports Medicine, Rothman Orthopaedic Institute, Philadelphia, USA

**Keywords:** flexibility, stretching, range of motion, posterior capsule, physical therapy, cross body stretch

## Abstract

Purpose

This study aimed to determine whether prone cross-body adduction (superman stretch) improves range of motion (ROM) more than the sleeper stretch.

Methods

Collegiate overhead athletes were randomized to either a sleeper group or a superman stretch group. ROM measurements were collected before and after stretches by three orthopedic surgeons.

Results

We assessed a total of 212 shoulders. Both stretches demonstrated significant improvements in ROM, except horizontal adduction, which only improved in the superman stretch group.

Conclusions

The superman stretch may be superior in producing immediate improvements in horizontal adduction when compared to the traditional sleeper stretch.

## Introduction

Throwing athletes subject their shoulders to repeated forces, which leads to several adaptive changes [1]. One response to this repeated activity is an increase in external rotation (ER). Increased ER is commonly balanced with a decrease in internal rotation (IR), maintaining an equivalent total rotational motion arc [2].

These adaptive changes act as the catalyst for pathology, as the majority of shoulder injuries occur at the limits of ER [1-2]. Repeated microtrauma leads to soft tissue and bony changes, reducing IR and leading to posterior shoulder tightness (PST) [3]. This also serves as a risk factor for developing injuries including internal shoulder impingement, superior labral tears, and partial-thickness rotator cuff tears, which may negatively impact athletic performance [4]. Furthermore, these adaptive changes can increase the risk of glenohumeral internal rotation deficit (GIRD), which is associated with significant shoulder dysfunction [5]. The majority of shoulder-related throwing injuries can be treated conservatively with strengthening and stretching [6]. While the data evaluating the efficacy of specific stretches is limited, it is acknowledged that stretching programs help maintain shoulder flexibility and improve the range of motion (ROM) of many joints [7].

The sleeper stretch is commonly used in throwing athletes, as it isolates the soft tissue restraints in the posterior shoulder. Laudner et al. investigated the use of the sleeper stretch in collegiate baseball players and found that it led to significant improvements in shoulder IR and horizontal adduction [8]. The stretch involves passive scapular stabilization with the patient’s body weight and IR of the shoulder while lying on the side. Another popular stretch for throwing athletes is the “cross-body stretch,” which involves horizontal adduction of the involved arm at 90° of flexion using the contralateral arm. McClure et al. found that this stretch resulted in greater IR improvement than the sleeper stretch in asymptomatic patients [9]. This technique has received some criticism, as it is performed without stabilization of the scapula. Prior research has suggested that scapular stabilization while performing stretches leads to significant improvements in ROM [10]. With these factors in mind, we modified the cross-body stretch to be performed in the prone position, termed the “superman stretch”. The prone positioning allows the stretching to be achieved through passive body weight rather than proper arm positioning and applied forces by the contralateral arm. To date, no study has evaluated the effects of the superman stretch on posterior shoulder flexibility and ROM. The primary aim of our study is to determine whether the use of superman stretch provides greater time-zero improvement in shoulder ROM than the sleeper stretch in a cohort of collegiate athletes. We hypothesize that the superman stretch would demonstrate similar or greater improvements in shoulder flexibility and ROM compared to the sleeper stretch.

## Materials and methods

Patient selection

Institutional Review Board approval was obtained by our institution. Asymptomatic, in-season division III collegiate overhead athletes, including baseball/softball, volleyball, and tennis, were enrolled prospectively and randomized at our institution. Exclusion criteria included patients with a history of prior surgery, evidence of hyperlaxity (defined as Beighton score of ≥6), or initial IR greater than 70°. Once enrolled, patients were randomized to either the sleeper stretch cohort or the superman stretch cohort, with both shoulders allocated to the same stretching protocol. Allocation was done using a computer-generated block randomization sequence, with the study examiners blinded to patient allocation. Due to the nature of the stretches, it was impossible to blind the patients to their allocation.

Measurements and instrumentation

Measurements were performed by one of three examiners, all of whom were fellowship-trained orthopedic surgeons, and certified athletic trainers were used for patient positioning and stabilization. The examiner used a digital goniometer (SHAHE Inc, Wenzhou, China) to measure shoulder IR and ER and glenohumeral horizontal adduction. For IR, the participant was examined in a supine position, with the shoulder and elbow in 90° of abduction and flexion, respectively, and with the humerus supported to ensure a neutral horizontal position (level with the acromion process). A certified athletic trainer stabilized the scapula by applying pressure to the anterior acromion, with the participant passively internally rotated until the termination of humeral rotation. The digital goniometer was aligned with the ulna, providing an angle between the forearm and a perpendicular plane to the examination table.

For ER, the participant was examined in the supine position, with the shoulder and elbow in 90° of abduction and flexion, respectively, and with the humerus supported to ensure a neutral horizontal position (level with the acromion process). A certified athletic trainer passively externally rotated the humerus, and with the other hand, stabilized the scapula by applying pressure to the anterior acromion until termination of humeral rotation. The digital goniometer was aligned with the ulna, providing an angle between the forearm and a perpendicular plane to the examination table.

For glenohumeral horizontal adduction, participants began in a supine position, with both shoulders flat against a standard examination table. A certified athletic trainer stood at the head of the examination table and positioned the test shoulder and elbow into 90° of abduction and flexion, respectively. The certified athletic trainer stabilized the lateral border of the scapula by providing a posteriorly directed force. With the other hand, the certified athletic trainer held the proximal portion of the participant’s forearm just distal to the elbow and passively moved the humerus into horizontal adduction, where the examiner then measured the amount of motion present. The digital goniometer was aligned with the ventral midline of the humerus to complete the measurement. The angle created by the end position of the humerus to 0° of horizontal adduction was recorded as total glenohumeral horizontal adduction motion.

Procedure descriptions

Before performing the randomized stretch, each participant had a baseline ROM assessed using the digital goniometer, as described above by one of the examiners. Following baseline measurements, participants were moved to a separate room and were supervised by a certified athletic trainer while performing their assigned stretch for three rounds of 30 s on each arm, with allocation and pre-stretch ROM measurements blinded to the examiner who is performing the measurements. After completing three rounds of stretches, participants were brought back to the examination room, where the same examiner performed the ROM measurements again using the digital goniometer. Both cohorts were provided the same but separate instruction by a certified athletic trainer and provided a picture demonstrating either the sleeper or superman stretch to provide a consistent description of the respective stretches. In addition, the stretch was demonstrated to the patients by the certified athletic trainer.

**Figure 1:**
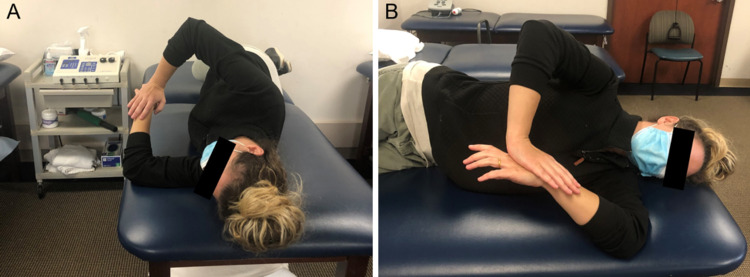
The sleeper stretch participants were placed in a side-lying position, with the scapula stabilized against the table. The elbow was placed in 90° of flexion and the participant’s contralateral arm was used to rotate the arm toward the table with maximum IR

**Figure 2:**
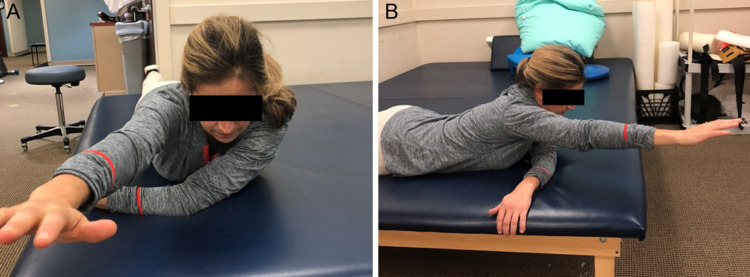
The superman stretch participants were placed prone on the athletic trainer’s table. The ipsilateral shoulder was flexed 90° and adducted horizontally across the torso, and the contralateral shoulder was flexed forward 180°

The sleeper stretch participants were placed in a side-lying position, with the scapula stabilized against the table. The elbow was placed in 90° of flexion, and the participant’s contralateral arm was used to rotate the arm toward the table with maximum IR. This position was held for 30 s. This stretch protocol was repeated three times for each side in succession, with rest for one side occurring, while the other was being stretched. The superman stretch participants were placed prone on the athletic trainer’s table. The ipsilateral shoulder was flexed 90° and adducted horizontally across the torso, and the contralateral shoulder was flexed forward 180°. This position was held for 30 s. This stretch protocol was repeated three times. The sleeper stretch and superman stretch can be seen in Figures 1-2, respectively.

Statistical analysis

Degrees of IR, ER, the total arc of motion, and horizontal adduction before and after stretching were reported as mean and standard deviations. Paired *t*-tests were conducted to determine whether there were any significant differences in baseline and post-stretch ROM values. Two-sample *t*-tests were carried out to determine whether there were any significant differences in ROM values for the sleeper stretch and superman stretch groups. Effect sizes for the comparative analysis were calculated using Hedges’ *g*-test. A *p*-value <0.05 was considered statistically significant. A power analysis found that 200 shoulders required enrollment to achieve a power of 80%.

## Results

**Figure 3:**
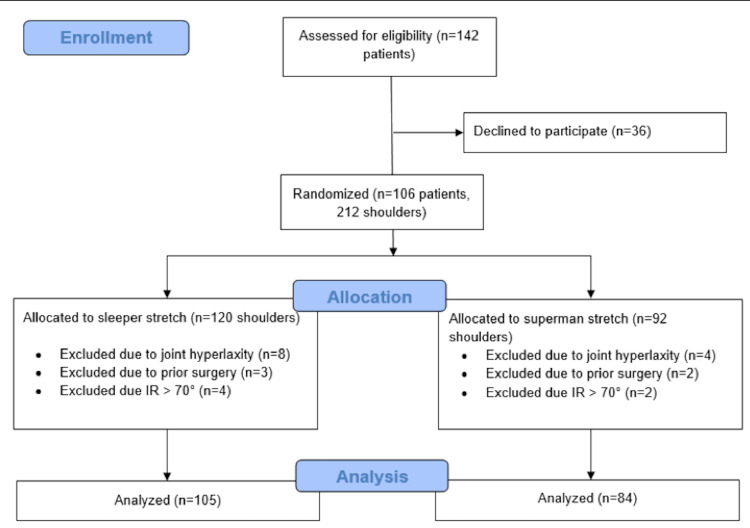
Participant enrollment, exclusion, and randomization

Patient enrollment, exclusion, and randomization can be seen in Figure 3. A total of 106 athletes (212 shoulders) were found to satisfy the inclusion criteria. Twelve shoulders were excluded from the study due to the presence of joint hyperlaxity, five were excluded due to prior surgery, and six were excluded due to an initial IR measurement >70°. A total of 189 shoulders were included for final data analysis, with 105 shoulders in the sleeper stretch group and 84 shoulders in the superman stretch group. The cohort included patients ranging from 18 to 23 years of age, all of whom played a sport for a division III collegiate program.

Standard errors of the mean (SEMs) for each ROM value were calculated using the recorded pre-stretch values. SEM values for IR, ER, the arc of motion, and horizontal adduction were 1.06º, 0.86º, 1.40º, and 0.70º, respectively. This translated to minimum detectable changes of 2.94º, 2.38º, 3.88º, and 1.94º, respectively. Table 1 describes the ROM measurements before and after stretching. Before stretching, there was only a significant difference between the sleeper and superman stretch groups for ER (sleeper: 89.5 vs. superman: 92.7, *p*=0.02). Significant increases in all ROM parameters included in the study were found for both groups, except for horizontal adduction in the sleeper stretch group (baseline: 46.1 vs. post-stretch: 48.2, *p*=0.08).

**Table 1:** Changes in ROM Before and After Stretching IR, internal rotation; ER, external rotation; ROM, range of motion. ROM before and after the sleeper and superman stretch interventions. *p*-Values are reported from paired-sample *t*-tests. *Statistically significant value (*p*≤0.05).

Variable	Pre-Stretch	Post-Stretch	Change in ROM	*p*-Value
Sleeper stretch				
IR	52.7 ± 15.2	60.1 ± 16.9	7.41	<0.001*
ER	84.7 ± 13.5	89.5 ± 12.0	4.81	0.007*
Arc of motion	137.4 ± 21.2	149.6 ± 22.6	12.22	<0.001*
Horizontal adduction	46.1 ± 8.8	48.2 ± 9.2	2.15	0.083
Superman stretch				
IR	48.7 ± 14.0	58.2 ± 15.5	9.54	<0.001*
ER	89.0 ± 10.1	92.7 ± 11.3	3.70	0.027*
Arc of motion	137.6 ± 17.3	150.9 ± 17.8	13.24	<0.001*
Horizontal adduction	48.4 ± 10.4	52.7 ± 10.8	4.27	0.010*

Table 2 describes the differences in post-stretch ROM between the two groups. The superman stretch group had a significantly higher degree of horizontal adduction than the sleeper stretch group (52.7° vs. 48.2°, *p*=0.002). This difference in horizontal adduction had a medium effect size (0.457). All other ROM measurements had small effect sizes between groups.

**Table 2:** Comparison of Post-Stretch ROM for the Sleeper and Superman Stretches IR, internal rotation; ER, external rotation; ROM, range of motion. Comparison of post-stretch ROM between the sleeper and superman stretch interventions. *p*-Values are reported from two-sample *t*-tests. *Statistically significant value (*p*≤0.05).

Variable	Sleeper Stretch	Superman Stretch	Effect Size	*p*-Value
IR	60.1 ± 16.9	58.2 ± 15.5	0.117	0.428
ER	89.5 ± 12.0	92.7 ± 11.3	0.274	0.064
Arc of motion	149.6 ± 22.6	150.9 ± 17.8	0.064	0.670
Horizontal adduction	48.2 ± 9.2	52.7 ± 10.8	0.457	0.002*

## Discussion

Athletes who perform repetitive overhead movements at the extremes of glenohumeral motion place immense repetitive stresses on the shoulder joint and soft tissues [3]. Pathological changes secondary to overhead throwing movements alter the athlete’s total arc of motion, reducing IR and increasing ER. The current prevailing theory is that osseous adaptations, PST, and thickening from microtrauma can result in glenohumeral passive ROM deficits with an increased risk of injuries to the shoulder. Generally, evidence suggests that GIRD and PST are associated with a shoulder injury in overhead athletes [11]. Deficits in ROM from GIRD and PST can be found in both symptomatic and asymptomatic athletes, with studies suggesting up to four times increased incidence of shoulder and elbow injuries compared with athletes with normal ROM [11]. Restoring ROM with stretching has been suggested to lower the rate of injury in overhead athletes [2]. Although a stretching protocol to optimize ROM and prevent injury has yet to be elucidated, studies have demonstrated that the sleeper stretch and cross-body stretch increase pos­­terior shoulder motion, IR, and horizontal adduction [8-9]. Shitara et al. prospectively evaluated high-school pitchers separated into stretching, strengthening, or no intervention groups and found that the incidence of injury was significantly lower in the stretching group [6]. This study suggested that post-activity stretching could mitigate cumulative pathophysiological changes, reduce glenohumeral ROM changes, and decrease the risk of injury. In conjunction with the current literature, our study provides additional evidence that stretching protocols can increase shoulder ROM in throwing athletes [12].

Our study investigated the effect of the superman stretch and sleeper stretch on ROM and found that both stretches led to significant time-zero ROM improvements. Only horizontal adduction was significantly improved by the superman stretch compared to the sleeper stretch. Our measurement technique demonstrated similar SEM values to prior studies utilizing similar stretches for IR, ER, the arc of motion, and horizontal adduction [9]. Previous studies have also validated this approach with high intra-rater (0.93-0.99) and inter-rater (0.63-0.77) reliability values [13]. We believe this demonstrates that our findings are likely due to significant changes in ROM, rather than measurement error.

We found that the sleeper stretch effectively increases IR, ER, and the total arc of motion, consistent with previous findings on the utilization of this stretch [8-9]. We further found that the sleeper stretch did not lead to significant improvements in horizontal adduction. This is concerning, as even small decreases in horizontal adduction have been associated with increased rates of injury [4]. Shanley et al. demonstrate that side-to-side differences in horizontal adduction >15º were associated with a four times greater risk of shoulder injury in adolescents [11]. These findings suggest that stretching protocols effective at minimizing horizontal adduction deficits are pivotal for throwing athletes. However, our findings contrast with Laudner et al. and Yamauchi et al., where horizontal adduction was improved following the sleeper stretch [8,14]. The discrepancy in these findings may be due to the difference in the technique used. Yamauchi et al. utilized a towel underneath the humerus to explicitly increase the amount of glenohumeral adduction. Laudner et al. utilized a clinician to apply passive IR and adduction during the sleeper stretch. In comparison, our study’s superman stretch allows for a simple technique that can be easily replicated without the need for supervision or assistance.

In our practice, we utilize the superman stretch due to its ability to maintain scapular stabilization passively using the patient’s body weight. This is similar to the bodyweight assistance provided in the sleeper stretch technique. With the superman stretch, once the arm is positioned, the patient’s only variable to consider is the degree of body rollover. In comparison, while performing the sleeper stretch, patients must consider proper arm positioning, degree of body rollover, and the proper application of IR force by the contralateral arm. These variables make this technique more difficult to perform without proper supervision. Additionally, the superman stretch resulted in greater horizontal adduction compared to the sleeper stretch in our study. This degree of improvement is likely to be clinically significant, as Camp et al. described that throwing athletes had a 4.5º horizontal adduction deficit and that this deficit may be associated with increased risks of injury [15].

We are aware of several limitations with our study design. Age and sex were not collected, so it is unclear whether there may have been any significant differences between the two groups. However, patients were randomized to each group, they were asymptomatic, and only one ROM value differed at baseline between groups. Hand dominance was not considered, as stretches were performed on both sides of the participants. Additionally, this study only evaluated ROM before and immediately after the performance of the stretch. For this reason, the results of this study cannot be used to make conclusions regarding the long-term effects of these stretches. However, we believe this helped minimize the bias introduced by external stretching programs or sports practices that would be impossible to control for in a long-term study. Finally, the ROM measurements were recorded at a single time point and were not re-assessed by a second examiner.

## Conclusions

In conclusion, our study demonstrated that both the superman and sleeper stretches led to significant immediate improvements in ROM. Furthermore, we found that the superman stretch may be superior in improving immediate horizontal adduction. The results suggest that the superman technique should be considered for stretching routines in overhead athletes.

## References

[1] Burkhart SS, Morgan CD, Kibler WB (2003). The disabled throwing shoulder: spectrum of pathology. Part I: pathoanatomy and biomechanics. Arthroscopy.

[2] Wilk KE, Macrina LC, Fleisig GS (2011). Correlation of glenohumeral internal rotation deficit and total rotational motion to shoulder injuries in professional baseball pitchers. Am J Sports Med.

[3] Crockett HC, Gross LB, Wilk KE (2002). Osseous adaptation and range of motion at the glenohumeral joint in professional baseball pitchers. Am J Sports Med.

[4] Myers JB, Laudner KG, Pasquale MR, Bradley JP, Lephart SM (2006). Glenohumeral range of motion deficits and posterior shoulder tightness in throwers with pathologic internal impingement. Am J Sports Med.

[5] Rose MB, Noonan T (2018). Glenohumeral internal rotation deficit in throwing athletes: current perspectives. Open Access J Sports Med.

[6] Shitara H, Yamamoto A, Shimoyama D (2017). Shoulder stretching intervention reduces the incidence of shoulder and elbow injuries in high school baseball players: a time-to-event analysis. Sci Rep.

[7] Reuther KE, Larsen R, Kuhn PD, Kelly JD 4th, Thomas SJ (2016). Sleeper stretch accelerates recovery of glenohumeral internal rotation after pitching. J Shoulder Elbow Surg.

[8] Laudner KG, Sipes RC, Wilson JT (2008). The acute effects of sleeper stretches on shoulder range of motion. J Athl Train.

[9] McClure P, Balaicuis J, Heiland D, Broersma ME, Thorndike CK, Wood A (2007). A randomized controlled comparison of stretching procedures for posterior shoulder tightness. J Orthop Sports Phys Ther.

[10] Salamh PA, Kolber MJ, Hanney WJ (2015). Effect of scapular stabilization during horizontal adduction stretching on passive internal rotation and posterior shoulder tightness in young women volleyball athletes: a randomized controlled trial. Arch Phys Med Rehabil.

[11] Shanley E, Rauh MJ, Michener LA, Ellenbecker TS, Garrison JC, Thigpen CA (2011). Shoulder range of motion measures as risk factors for shoulder and elbow injuries in high school softball and baseball players. Am J Sports Med.

[12] Mine K, Nakayama T, Milanese S, Grimmer K (2017). Effectiveness of stretching on posterior shoulder tightness and glenohumeral internal-rotation deficit: a systematic review of randomized controlled trials. J Sport Rehabil.

[13] Kevern MA, Beecher M, Rao S (2014). Reliability of measurement of glenohumeral internal rotation, external rotation, and total arc of motion in 3 test positions. J Athl Train.

[14] Yamauchi T, Hasegawa S, Nakamura M (2016). Effects of two stretching methods on shoulder range of motion and muscle stiffness in baseball players with posterior shoulder tightness: a randomized controlled trial. J Shoulder Elbow Surg.

[15] Camp CL, Cancienne JM, Degen RM, Dines JS, Altchek DW, Werner BC (2017). Factors that increase the risk of infection after elbow arthroscopy: analysis of patient demographics, medical comorbidities, and steroid injections in 2,704 Medicare patients. Arthroscopy.

